# 353. Outpatient parenteral antimicrobial therapy (OPAT) in managing vertebral osteomyelitis

**DOI:** 10.1093/ofid/ofad500.424

**Published:** 2023-11-27

**Authors:** Evelyn Villacorta Cari, Michael Young, Alisha Clemons, Ashley Logan, Ryan Mynatt, Jaime Soria

**Affiliations:** University of Kentucky, Lexington, Kentucky; University of Kentucky, Lexington, Kentucky; University of Kentucky, Lexington, Kentucky; University of Kentucky HealthCare, Lexington, Kentucky; University of Kentucky, Lexington, Kentucky; University of Kentucky, Lexington, Kentucky

## Abstract

**Background:**

The outpatient parenteral antimicrobial therapy (OPAT) program was formally established at the University of Kentucky in 2019 to coordinate and monitor home-delivered antimicrobial treatment. This analysis describes the epidemiologic, microbiologic features, and outcomes of subjects diagnosed with vertebral osteomyelitis referred to the OPAT program.

**Methods:**

A cross-sectional study was conducted; we analyzed the data collected during the OPAT program evaluation from July 2019 to October 2022. The data were collected and managed using REDCap electronic data capture tools hosted at the University of Kentucky.

**Results:**

The analysis included 411 subjects diagnosed with vertebral osteomyelitis. referred to the OPAT program. The median age was 51 years old (range: 22-91), and 254 (61.8%) were male. Active or previous history of intravenous drug use (IVDU) was reported in 193 (47.1%), being the most recent use within the last 30 days reported in 142 (74.0%). Admission to the intensive care unit (ICU) was documented in 110 (27.0%) subjects; mainly due to infection related to the OPAT indication; the median Charlson Comorbidity Index score was 2 (IQR: 0-4). The median duration in the OPAT program was 18 (IQR: 11-34) days. The distribution by spinal segment was as follows 115 (28.0%) in the lumbar spine, 75 (18.2%) in the thoracic spine, 58 (14.1%) in the cervical spine, 57 (13.9%) in the sacral and sacrococcygeal spine, more than two segments in 12 (2.0%) subjects, and unspecified in 94 (22.9%) subjects. The most common pathogen isolated was *Staphylococcus aureus* in 182 (44.3%) subjects, followed by *Streptococcus* spp in 19 (4.6%). Among gram-negative bacterium, *Serratia* spp and *Pseudomonas aeruginosa* were the more prevalent pathogens, 18 (4.4%) and 15 (3.6%), respectively, mainly when the sacrococcygeal spinal segment was compromised. Fungal infection was documented in 13 (3.1%) subjects. The most common antimicrobial used was daptomycin, followed by vancomycin in 97 (23.6%) and 78 (19.0%) subjects, respectively. In this cohort, , the overall mortality rate was 2.2%.

**Conclusion:**

The OPAT program is a convenient and safe alternative for treating vertebral osteomyelitis that requires a prolonged course of intravenous antimicrobial treatment.

**Disclosures:**

**All Authors**: No reported disclosures

